# Comparing Multipollutant Emissions-Based Mobile Source Indicators to Other Single Pollutant and Multipollutant Indicators in Different Urban Areas

**DOI:** 10.3390/ijerph111111727

**Published:** 2014-11-14

**Authors:** Michelle M. Oakes, Lisa K. Baxter, Rachelle M. Duvall, Meagan Madden, Mingjie Xie, Michael P. Hannigan, Jennifer L. Peel, Jorge E. Pachon, Siv Balachandran, Armistead Russell, Thomas C. Long

**Affiliations:** 1 National Center for Environmental Assessment, U.S. Environmental Protection Agency, Research Triangle Park, NC 27711, USA; E-Mails: meagankmadden@gmail.com (M.M.); long.tom@epa.gov (T.C.L.); 2 Oak Ridge Institute for Science and Education, Oak Ridge, TN 37831, USA; E-Mail: mmoakes17@gmail.com; 3 National Exposure Research Laboratory, U.S. Environmental Protection Agency, Research Triangle Park, NC 2711, USA; E-Mails: baxter.lisa@epa.gov (L.K.B.); duvall.rachelle@epa.gov (R.M.D.); 4 Department of Mechanical Engineering, College of Engineering and Applied Science, University of Colorado-Boulder, Boulder, CO 80309, USA; E-Mails: mingjie.xie@colorado.edu (M.X.); hannigan@colorado.edu (M.H.); 5 Department of Civil and Environmental Engineering, Virginia Polytechnic Institute and State University, Blacksburg, VA 24061, USA, E-Mail: mingjie1@vt.edu; 6 Department of Environmental and Radiological Health Sciences, Colorado State University, Fort Collins, CO 80523, USA; E-Mail: jennifer.peel@colostate.edu; 7 Program of Environmental Engineering, Universidad de La Salle, Bogota, CO 111711, USA; E-Mail: jpachon@unisalle.edu.co; 8 Department of Civil & Environmental Engineering, Georgia Institute of Technology, Atlanta, GA 30332, USA; E-Mails: siv@gatech.edu (S.B.); ted.russell@ce.gatech.edu (A.R.); 9 Union of Concerned Scientists, Washington, DC 20006, USA

**Keywords:** multipollutant, air pollution, exposure metrics, source apportionment, mobile sources, emissions-based indicators

## Abstract

A variety of single pollutant and multipollutant metrics can be used to represent exposure to traffic pollutant mixtures and evaluate their health effects. Integrated mobile source indicators (IMSIs) that combine air quality concentration and emissions data have recently been developed and evaluated using data from Atlanta, Georgia. IMSIs were found to track trends in traffic-related pollutants and have similar or stronger associations with health outcomes. In the current work, we apply IMSIs for gasoline, diesel and total (gasoline + diesel) vehicles to two other cities (Denver, Colorado and Houston, Texas) with different emissions profiles as well as to a different dataset from Atlanta. We compare spatial and temporal variability of IMSIs to single-pollutant indicators (carbon monoxide (CO), nitrogen oxides (NO_x_) and elemental carbon (EC)) and multipollutant source apportionment factors produced by Positive Matrix Factorization (PMF). Across cities, PMF-derived and IMSI gasoline metrics were most strongly correlated with CO (*r* = 0.31–0.98), while multipollutant diesel metrics were most strongly correlated with EC (*r* = 0.80–0.98). NO_x_ correlations with PMF factors varied across cities (*r* = 0.29–0.67), while correlations with IMSIs were relatively consistent (*r* = 0.61–0.94). In general, single-pollutant metrics were more correlated with IMSIs (*r* = 0.58–0.98) than with PMF-derived factors (*r* = 0.07–0.99). A spatial analysis indicated that IMSIs were more strongly correlated (*r* > 0.7) between two sites in each city than single pollutant and PMF factors. These findings provide confidence that IMSIs provide a transferable, simple approach to estimate mobile source air pollution in cities with differing topography and source profiles using readily available data.

## 1. Introduction

A large body of evidence is now available on the health effects of individual air pollutants [1,2,3,4,5,6]. However, limited research has been conducted on the health effects of air pollution mixtures. People are not exposed separately to individual ambient-generated air pollutants, but rather simultaneously to mixtures of pollutants from multiple sources. In order to gain a comprehensive understanding of public health impacts of ambient air pollution, leading researchers and institutions have called for additional investigations into the health effects of air pollution mixtures [7,8,9,10].

A key challenge in multipollutant research is defining mixtures of interest and developing quantitative exposure metrics of those mixtures. Many approaches exist, based on co-emission from common sources, common chemical properties, or common biological modes of action, which individually address different research questions [11]. From an air quality management standpoint, identifying and estimating multipollutant source mixtures responsible for health effects is beneficial, and estimating impacts from ubiquitous sources, such as traffic pollution, is of particular interest. Mobile source pollution has been associated with multiple health effects, including respiratory and cardiovascular morbidity [5,12,13]. In addition, many people live near roadways or spend time daily commuting to work or school, resulting in frequent and sustained exposures to traffic pollution.

Exposure to mobile sources has been estimated using a variety of metrics, which can be either single pollutant or multipollutant in nature. The simplest quantitative approach for estimating traffic pollution is to choose a single pollutant “tracer” that is elevated in source emissions, such as black carbon (BC) for diesel exhaust [14]. The advantages of using a tracer include data availability, transparency, and direct links to epidemiologic and toxicological studies using that pollutant. However, disadvantages include emissions from other sources, pollutant transformation in the atmosphere, and monitoring sites impacted by local conditions that can make it difficult to link a pollutant to an intended source.

In an effort to more directly identify mobile source impacts, investigators have employed more sophisticated metrics that incorporate data on multiple pollutants, such as source metrics/factors derived from source apportionment techniques. Source apportionment is typically used to resolve a series of factors or “source profiles” and estimate their contribution to the ambient air pollution mixture [15,16,17,18]. By incorporating additional data from multiple pollutants, source apportionment increases the amount of information in the metric, potentially improving the ability to resolve multiple factors simultaneously, including source factors related to mobile source pollution. Disadvantages of source apportionment include the requirement for a substantial amount of data and complicated processing; in addition, source factors can be difficult to interpret, have higher daily variability than indicated emissions inventories, or poor spatial representativeness across urban geographical scales. An additional limitation specific to mobile source apportionment is the difficulty in resolving gasoline and diesel pollution mixtures [19,20].

To address some limitations inherent with source apportionment of traffic pollution, Pachon *et al.* [21] developed a series of Integrated Mobile Source Indicators (IMSI) and assessed their performance in Atlanta, Georgia. The IMSIs combine readily available concentration data of key mobile source pollutants (CO, NO_x_, EC) with emissions estimates for gasoline, diesel, and total vehicles. In Atlanta, Pachon *et al.* (2012) showed that IMSIs provide additional information to build a more robust metric than a single pollutant concentration while providing a more stable approach for estimating temporal and urban scale trends in traffic pollution than source apportionment. Such results reflect the promise of using IMSIs to estimate traffic in other cities in future studies.

In this research we extend the application of a variety of IMSIs to other geographical locations and compare their generalizability and transferability (to other cities) with single pollutant indicators and source apportionment factors. Urban areas vary in terms of the main sources of air pollution, traffic patterns, geographic location and climate, topography, and land use. These differences may affect the extent to which different mobile source indicators represent exposure to traffic emissions. Comparison of these metrics among different cities can provide information useful in selecting mobile source indicators for health studies as well as serve as a model for constructing and selecting multipollutant metrics for other sources.

## 2. Methods

### 2.1. Urban Areas

Daily mobile source metrics were calculated at a central site and a secondary site in three urban areas with contrasting emissions (Atlanta, Denver and Houston). Urban areas were selected based on (1) availability of air quality data at multiple monitoring sites within urban boundaries, (2) contrasting mobile source emission profiles as defined by the 2008 National Emissions Inventory [22], and (3) diverse differences in topography and meteorology.

Briefly, Atlanta, Georgia is an urban area located in the southeastern U.S. with major air pollution sources including motor vehicles, electricity generating units (EGUs) and biogenic sources (e.g., vegetation). Pollution in Atlanta is affected by high relative humidity and frequent stagnant weather conditions during the summer. Denver, Colorado is a high-elevation urban area located in the Rocky Mountains largely impacted by emissions from motor vehicles, with regional influences from energy development and biogenic sources, including forest fires. Ambient air pollution in Denver is affected by meteorological patterns and topographical features associated with mountainous regions. Houston, Texas is an urban, industrial, port city located in the mid-southwestern U.S. with air pollution from a combination of mobile sources (e.g., on-road and shipping) and petrochemical plants. Due to Houston’s coastal location, ambient pollution is largely influenced by meteorological dynamics of oceanic regions (e.g., the land-sea breeze).

### 2.2. Air Quality Data

In each urban area, different monitoring networks were used to retrieve air pollution data at a central-site monitor representing urban-scale exposure and a secondary monitor located within urban boundaries. The monitoring network, sampling sites, and sampling period used in this study are summarized in Table 1. For all three cities, data were obtained from either the Air Quality System (AQS) data repository supported by the U.S. Environmental Protection Agency (EPA) or from local field campaigns or networks, such as the SouthEastern Aerosol CHaracterization (SEARCH) study [23] in Atlanta, GA and the Denver Aerosol Sources and Health (DASH) study in Denver, CO.

Central-site monitors in this study were the Jefferson Street site in Atlanta, GA, the Palmer site in Denver, CO, and the Aldine site in Houston, TX (Table 1). Methods for air pollution monitoring and quality assurance protocols supported by the SEARCH (for Atlanta measurements) and DASH (for Denver measurements) networks are discussed in detail in Hansen *et al.* [23] and Vedal *et al.* [24] respectively, but are also presented in Table 1. Ambient air quality data at the Aldine monitoring site in Houston, TX were retrieved using the EPA AQS database. At AQS sites, NO_x_ (defined as the sum of nitric oxide (NO) and nitrogen dioxide (NO_2_)) was measured using a chemiluminesence technique coupled to a molybdenum oxide substrate, which directly measures NO. Using this technique, ambient NO_2_ is reduced to NO by a molybdenum oxide substrate and subsequently measured by chemiluminesence. AQS carbon monoxide (CO) was measured using a nondispersive infrared (NDIR) detection technique. AQS fine particle (PM_2.5_, *i.e.*, particles less than 2.5 μm in diameter) elemental carbon (EC) collected on quartz filter substrates (over a 24-h time interval) was quantified using thermal optical reflectance (TOR). Additionally, PM_2.5_ ionic and elemental species were used for source apportionment analysis. AQS filter-based ionic species (SO_4_^2^^−^, NO_3_, NH_4_^+^) collected on a Teflon substrate were extracted in deionized water and analyzed by ion chromatography. AQS PM_2.5_ elemental species (e.g., Al, Cu, Fe) were measured using X-ray fluorescence.

**Table 1:** Monitoring networks.

Urban Area	Site	County	Study Period	Monitoring Network	Measurement Methods
Atlanta, GA	Jefferson Street (JST) *Central-site*	Fulton (JST)	2005–2010	SEARCH	CHL (NO_x_)
					NDIR (CO)
					TOR (EC, BC, OC)
					IC (ions)
					AC (NH_4_^+^)
					XRF (trace elements)
Atlanta, GA	South Dekalb (SD) *Secondary Site*	Dekalb (SD)	2005–2010	AQS	CHL (NO_x_) *
					NDIR (CO)
					TOR (EC)
					IC (ions)
					XRF (trace elements)
Denver, CO	Palmer (PAL) * Central-site	Denver (PAL)	2004–2005	AQS	CHL (NO_x_) **
					NDIR (CO)
					TOT (EC,OC)
					IC (ions)
Denver, CO	Alsup (ALS) ** Secondary Site	Adams (ALS)	2004–2005	AQS	CHL (NO_x_) *
					NDIR (CO)
					TOR (EC)
					IC (ions)
					XRF (trace elements)
Houston, TX	Aldine (AL) *Central-site*	Houston (AL)	2003–2005	AQS	CHL (NO_x_) *
					NDIR (CO)
					TOR (EC)
					IC (ions)
					XRF (trace elements)
Houston, TX	Deer Park (DP) *Secondary Site*	Houston (DP)	2003–2005	AQS	CHL (NO_x_) *
					NDIR (CO)
					TOR (EC)
					IC (ions)
					XRF (trace elements)

Notes: AQS: Air Quality System data repository operated by U.S. Environmental Protection Agency [25]; SEARCH: SouthEastern Aerosol Characterization study [23,26] ; DASH: Denver Aerosol Sources and Health Study [24,27,28],CHL: Chemiluminesence; NDIR Nondispersive Infrared Detection; TOR: Total Optical Reflectance; TOT: Total Optical Transmittance; IC: Ion Chromatography; XRF: X-ray Fluorescenc; * Gaseous data retrieved at AQS Camp site (AQS ID: 80310002); ** Gaseous data retrieved at AQS Welby site (AQS ID: 8013001).

Secondary site monitors were located at the South Dekalb site in Atlanta, Georgia, the Welby site in Denver, Colorado, and the Deer Park site in Houston, Texas. Ambient concentration data from secondary site monitors were obtained from the AQS database; thus, chemiluminesence, NDIR, and TOR techniques are employed for NO_x_, CO, and EC measurements, respectively. Gaseous pollutants (NO_x_ and CO) were generally reported as hourly averages then aggregated to a daily 1-h max concentration (maximum 1-h concentration observed in a 24-h time interval), whereas filter-based PM species were reported either every day or every 3rd or 6th day as a 24-h average concentration.

### 2.3. Single Pollutant Metrics

Single pollutant metrics included daily estimates of typical traffic related pollutants, including 1-h max nitrogen oxides (NO_x_), 24-h avg filter-based elemental carbon (EC), and 1-h max carbon monoxide (CO). The averaging time used for gaseous metrics (NO_x_ and CO) was selected to be more representative of short-duration, high-concentration exposures that induce health effects in controlled human exposure studies (e.g., Allred *et al.* [29]; Folinsbee [2]). However, similar results would be expected if 24-h avg values were used, since the correlations between the two daily metrics are relatively high (greater than 0.8 for CO, approximately greater than 0.86 for NO_x_) and the CVs are similar (Table 2).

### 2.4. Multipollutant Metrics: Source Apportionment Factors

Two types of multipollutant metrics were quantified for mobile sources: source apportionment factors resolved by Positive Matrix Factorization (PMF) [30] and emission-based integrated mobile source indicators (IMSI) [21]. Both PMF factors and IMSIs were calculated to represent diesel, gasoline, and total (diesel + gasoline) mobile source pollution.

Source apportionment results were provided by investigators at Georgia Institute of Technology for Atlanta [31], the University of Colorado-Boulder for Denver [32], and the U.S. EPA for Houston (refer to Supplemental Information). Due to large differences among air quality and data availability in the cities, different input datasets and versions of PMF were used to resolve mobile source factors for gasoline, diesel and total (gasoline + diesel) vehicles (Table 3); however, investigators attempted to maintain consistency in PMF analyses in different cities. In Atlanta and Houston, a combination of elemental, ionic, and carbonaceous (including elemental and organic) PM_2.5_ species were used as input data. In Denver, gaseous mobile source tracers (CO, NO_x_) as well as several PM_2.5_ ionic and carbonaceous species were used. Including additional elemental species to source apportionment analyses in Denver made it challenging to consistently resolve mobile source pollution [32]. Bootstrapping techniques and alternate uncertainty analysis were also performed to ensure data consistency.

**Table 2:** Distribution of central-site single pollutant and multipollutant metrics during 2005 in each urban Area.

Urban Area	Avg	SD	CV	Min	10	25	50	75	90	100	N
*1-h max* NO_x_*^+^*, *ppb* *(24 h avg.)*											
***Atlanta***	89.2	58.8	0.66	15.9	25.4	41.6	75.0	128.1	170.5	305.5	257
	(38.7)	(27.2)	(0.70)	(7.0)	(15.1)	(20.6)	(31.4)	(47.8)	(71.4)	(169.1)	(257)
***Denver ^+^***	42.0	11.3	0.27	15.0	27.0	33.0	42.0	50.0	57.0	81.0	260
	(25.2)	(9.1)	(0.36)	(4.2)	(13.0)	(18.2)	(25.7)	(32.0)	(40.0)	(50.0)	(260)
***Houston***	62.3	49.9	0.80	4.0	20.0	30.0	47.0	79.0	130.0	327.0	348
	(21.4)	(15.0)	(0.70)	(0.6)	(9.3)	(12.7)	(17.1)	(25.2)	(38.1)	(112.1)	(348)
*1-h max CO, ppm* *(24 h avg.)*											
***Atlanta***	0.86	0.65	0.76	0.17	0.31	0.39	0.65	1.06	1.87	4.09	365
	(0.44)	(0.22)	(0.55)	(0.13)	(0.22)	(0.27)	(0.33)	(0.47)	(0.67)	(1.82)	(365)
***Denver***	0.73	1.45	1.99	0.40	0.70	1.00	1.20	1.70	2.50	4.60	363
	(0.70)	(0.26)	(0.37)	(0.31)	(0.47)	(0.53)	(0.64)	(0.79)	(1.08)	(1.94)	(363)
***Houston***	0.88	0.48	0.55	0.00	0.40	0.50	0.70	1.10	1.64	2.80	365
	(0.45)	(0.15)	(0.33)	(0.00)	(0.31)	(0.36)	(0.43)	(0.52)	(0.63)	(1.19)	(365)
***EC***, μg/m^3^											
***Atlanta***	1.49	0.94	0.63	0.21	0.55	0.84	1.26	1.96	2.70	6.63	350
***Denver***	0.51	0.30	0.59	0.04	0.22	0.32	0.47	0.61	0.82	2.09	272
***Houston***	0.72	0.44	0.61	0.01	0.24	0.46	0.66	0.91	1.24	3.25	101
IMSIGV *											
***Atlanta***	1.5	1.0	0.67	0.3	0.5	0.7	1.2	2.0	3.0	5.4	257
***Denver***	2.2	0.6	0.30	0.7	1.5	1.7	2.1	2.5	3.0	4.9	260
***Houston***	1.5	0.9	0.60	0.3	0.7	0.9	1.3	1.8	2.9	5.6	335
IMSIDV *											
***Atlanta***	1.5	0.9	0.58	0.3	0.6	0.8	1.3	2.0	2.8	5.4	244
***Denver***	2.1	0.8	0.36	0.7	1.3	1.6	2.0	2.5	3.0	6.0	257
***Houston***	0.8	0.5	0.62	0.1	0.3	0.5	0.7	1.0	1.5	3.9	98
IMSIEB *											
***Atlanta***	1.5	0.9	0.60	0.3	0.6	0.8	1.3	2.0	2.8	5.3	244
***Denver***	2.1	0.7	0.31	0.7	1.3	1.6	2.0	2.4	2.9	5.3	257
***Houston***	1.5	0.9	0.57	0.5	0.7	0.9	1.3	1.9	2.7	6.1	94
*PMFGV*, μg/m^3^											
***Atlanta***	1.4	1.1	0.75	−0.3	0.5	0.8	1.1	1.8	2.8	7.6	344
***Denver***	0.2	0.1	0.44	0.0	0.1	0.1	0.2	0.3	0.3	0.7	272
***Houston***	3.5	2.0	0.58	−0.7	1.1	2.2	3.6	4.7	6.0	13.0	92
*PMFDV*, μg/m^3^											
***Atlanta***	2.3	2.0	0.84	−0.5	0.4	0.9	2.0	3.1	4.8	13.6	344
***Denver***	1.1	0.8	0.71	0.0	0.3	0.6	0.9	1.4	1.9	5.0	272
***Houston***	1.1	0.8	0.75	−0.2	0.3	0.6	0.9	1.5	2.1	5.7	92
*PMFMB*, μg/m^3^											
***Atlanta***	3.8	2.6	0.69	0.4	1.3	1.9	3.1	4.8	7.2	21.2	344
***Denver***	1.3	0.8	0.61	0.1	0.5	0.8	1.2	1.6	2.1	5.3	272
***Houston***	4.6	2.5	0.54	0.0	1.9	3.1	4.3	5.9	7.3	18.7	92

Notes: **^+^** Measured as NO_2_; * In arbitrary units.

**Table 3:** Source apportionment results in Atlanta, Denver, and Houston.

Urban Area (Monitoring Site)	PMF Version	Input Species	Factor (% Contribution of Pollutant Mass)
Atlanta (Jefferson Street)	PMF3.0	SO_4_^2−^, NO_3_^−^, NH_4_^+^, EC, OC1, OC2, OC3, OC4, OP, Al, Br, Ca, Cu, Fe, K, Mn, Pb, Se, Si, Zn	***Diesel vehicle (10.8%)***
			***Gasoline vehicle (14.9%)***
			Zinc (1.8%)
			Dust (1.6%)
			Sec NH_4_^+^ (17.8%)
			Biomass Burning (6.6%)
			NO_3_^−^ (6.8%)
			SO_4_^2−^, NH4^+^ (39.6%)
Denver (Palmer)	PMF2	NO_3_^−^, SO_4_^2−^, EC, OC, CO, NO_2_	***EC/Diesel (19.6%)***
			***Trace Gas/Gasoline (5.5%)***
			NO_3_^−^ (15.3%)
			SO_4_^2−^ (22.4%)
			OC (37.2%)
Houston (Aldine)	PMF5.0	SO_4_^2−^, NH_4_^+^, EC. OC1, OC2, OC3, OC4, Al, Br Ca, Cu, Fe, K, Mn, Pb, Se, Si, Zn	***Diesel (7.7%)***
			***Gasoline (25%)***
			Zinc-rich (0.6%)
			Dust/soil (12.2%)
			SO_4_^2−^ (38.6%)
			Biomass Burning (16%)

### 2.5. Multipollutant Indicators: Emission-Based Integrated Mobile Source Indicators (IMISI)

Multipollutant, emission-based integrated mobile source indicators (IMSI) were calculated using a similar approach to Pachon *et al.* [21]. This approach is designed to estimate mobile source pollution by combining ambient concentrations of traffic-related pollutants (CO, NO_x_, EC) with information on their annual mobile source emissions. Pollutant averaging times used in the IMSIs included the daily 1-h maximum concentration of gaseous pollutants (CO, NO_x_) and the 24-h average of filter-based EC measurements. The following three equations represent IMSIs for pollution of total mobile (gasoline + diesel) (IMSIEB) Equation (1), diesel mobile (IMSIDV) Equation (2), and gasoline mobile (IMSIGV) Equation (3) sources:

(1) $$IMSI_{EB}=\alpha_{\text{EC}}C_{EC}^{\prime }+\alpha_{{\text{NO}}_{\text{X}}}C_{NOx}^{\prime }+\alpha_{\text{CO}}C_{CO}^{\prime }$$

(2) $$IMSI_{DV}=\alpha_{\text{DV},\text{EC}}C_{EC}^{\prime }+\alpha_{\text{DV},{\text{NO}}_{\text{X}}}C_{NOx}^{\prime }$$

(3) $$IMSI_{GV}=\alpha_{\text{GV},\text{CO}}C_{CO}^{\prime }+\alpha_{\text{GV},{\text{NO}}_{\text{X}}}C_{NOx}^{\prime }$$

where α_ij_ is the normalized fraction of the emissions (E) of species j attributed to source i, e.g.,

$$\alpha_{\text{EB},\text{EC}=}\frac{\frac{E_{EC,mobile}}{E_{EC,total}}}{\frac{E_{EC,mobile}}{E_{EC,total}}+\frac{E_{NOx,mobile}}{E_{NOx,total}}+\frac{E_{CO,mobile}}{E_{CO,total}}}$$

and

$$\alpha_{\text{DV},\text{EC}=}\frac{\frac{E_{EC,diesel}}{E_{EC,total}}}{\frac{E_{EC,mobile}}{E_{EC,total}}+\frac{E_{NOx,mobile}}{E_{NOx,total}}}$$

and

$$\alpha_{\text{GV},\text{CO}=}\frac{\frac{E_{CO,gasoline}}{E_{CO,total}}}{\frac{E_{CO\;mobile}}{E_{CO,total}}+\frac{E_{NOx,mobile}}{E_{NOx,total}}}$$

and *C_j_’* is the normalized concentration of species j, *C_j_’* = *C_j_/σ_Cj_*.

Each IMSI is a 2 to 3 pollutant mixture used to represent different types of mobile source pollution. Total mobile source pollution is defined by a mixture of NO_x_, CO, and EC (Equation (1)), whereas diesel and gasoline pollution are each defined by a mixture of two pollutants (Equations (2) and (3)). To combine pollutants of different magnitudes, ambient concentrations were normalized (C_j_’) by the standard deviation of their daily concentration observed during the entire sampling period (σ_Cj_). Mobile source fractions (α_X_) were defined for each pollutant (CO, NO_x_, EC) by averaging monthly mobile source emissions in each urban area during 2007. For example, month-to-month variations in CO emissions were used to estimate CO mobile source fractions (α_X_) attributed to total, gasoline and diesel vehicles. Average mobile source fractions (α_X_) were applied over the entire sampling period for each pollutant and did not change on a daily basis.

Emission estimates and uncertainties for IMSIs were obtained from a number of sources. Annual, county-level mobile source emissions were estimated using the 2008 National Emissions Inventory (NEI) [22] developed by the U.S. EPA. The 2008 NEI reports annual emissions of criteria and hazardous air pollutants from a variety of anthropogenic and biogenic sources during 2008. Since EC traffic emissions are not included in the 2008 NEI, county-level annual emissions were estimated by averaging daily EC mobile source contributions during 2007 using SMOKE (Sparse Matrix Operator Kernel Emissions). The fraction of CO, NO_x_, and EC from diesel and gasoline mobile sources were estimated using the MOtor Vehicle Emissions Simulator model (MOVES2010). MOVES2010 was also used to quantify the uncertainty in annual traffic emissions associated with CO, NO_x_, and EC by evaluating month-to-month variation in total mobile source emissions. The standard deviation of the monthly median of mobile source emissions was defined as the uncertainty in annual mobile source emissions (shown in Figure S1).

### 2.6. Spatial and Temporal Comparison of Metrics in Different Urban Locations

The goal of this study is to apply IMSIs to different urban locations and compare them to other single pollutant and multipollutant traffic metrics within each location. To address this goal, single pollutant and multipollutant indicators were initially constructed in Atlanta, Denver, and Houston. In each city, Pearson correlation coefficients were used to compare day-to-day temporal trends among single pollutant and multipollutant mobile source indicators at a central site. In an inter-site spatial analysis, each metric was compared between a central site and a secondary site using Pearson correlation coefficients. Comparing temporal and spatial trends across metrics provides insight into how each metric characterizes variability in traffic pollution, which in turn, provides some information on exposure error.

## 3. Results

### 3.1. Concentrations of Central-Site Single Pollutant and Multipollutant Metrics

Table 2 shows the distributions of ambient values of central-site, single pollutant and multipollutant metrics, demonstrating variations in metrics by urban location. For single pollutants, the range observed in ambient concentrations was relatively similar across cities, with the exception of CO in Denver (median CO = 1.2 ppm) and EC in Atlanta (median EC = 1.3 ug/m^3^), which exhibited higher median concentrations compared to values in other cities. Multipollutant metrics demonstrated larger differences across cities. For example, average and median IMSI values were higher in Denver compared to other cities, while PMF values in Denver were the lowest observed across all cities.

When comparing day-to-day changes among different metrics, single pollutants tended to exhibit more temporal variation than multipollutant metrics. The coefficient of variation (CV) (standard deviation normalized by the mean) was generally the largest for single pollutant indicators, with generally larger CVs for daily 1-h max metrics (CO, NO_x_) compared to 24-h average metrics. Lower CVs were observed among multipollutant metrics (Table 2), with IMSIs generally showing the lowest CVs. A notable example of this trend is demonstrated in gasoline-related indicators (CO, PMFGV, IMSIGV); in particular, CVs in Denver were 1.99, 0.44, and 0.30 for 1-h max CO, PMFGV, and IMSIGV, respectively. Larger CVs for single pollutants indicate more day-to-day variability with respect to multipollutant metrics.

On a weekend/weekday basis, both single pollutant and multipollutant metric values decreased during the weekend in every location, which follows typical urban traffic patterns showing increased activity during the weekdays due to workplace commuting (not shown). Interestingly, NO_x_ and EC decreased more than CO during the weekend, consistent with the idea that diesel vehicle emissions drop more than gasoline vehicle emissions during the weekend. Large weekend decreases were simultaneously observed in diesel multipollutant metrics (emissions-based diesel indicator: IMSIDV and PMF-derived diesel factor: PMFDV) and to some extent in multipollutant total mobile source indicators (emissions-based total mobile indicator: IMSIEB and PMF-derived total mobile factor: PMFMB).

### 3.2. Characteristics of Multipollutant Metrics in Different Urban Locations

#### 3.2.1. Source Apportionment Factors

As demonstrated in Table 3, source apportionment results were different across cities, particularly in the number of resolved factors and mass attributed to different types of mobile source pollution (gasoline *vs.* diesel). In each urban area, source apportionment analyses yielded between 5 to 7 source factors, 2 of which corresponded to the gasoline and diesel fraction of mobile source pollution (Table 3). Other significant source factors identified in urban locations were secondary sulfate, nitrate, industrial sources, biomass burning, and crustal material. Additionally, in every city, the factor associated most closely with diesel sources comprised less than 20% of reconstructed PM_2.5_ mass; where the factor associated with gasoline sources accounted for less than 25% of PM_2.5_ mass in the urban areas.

**Figure 1:**
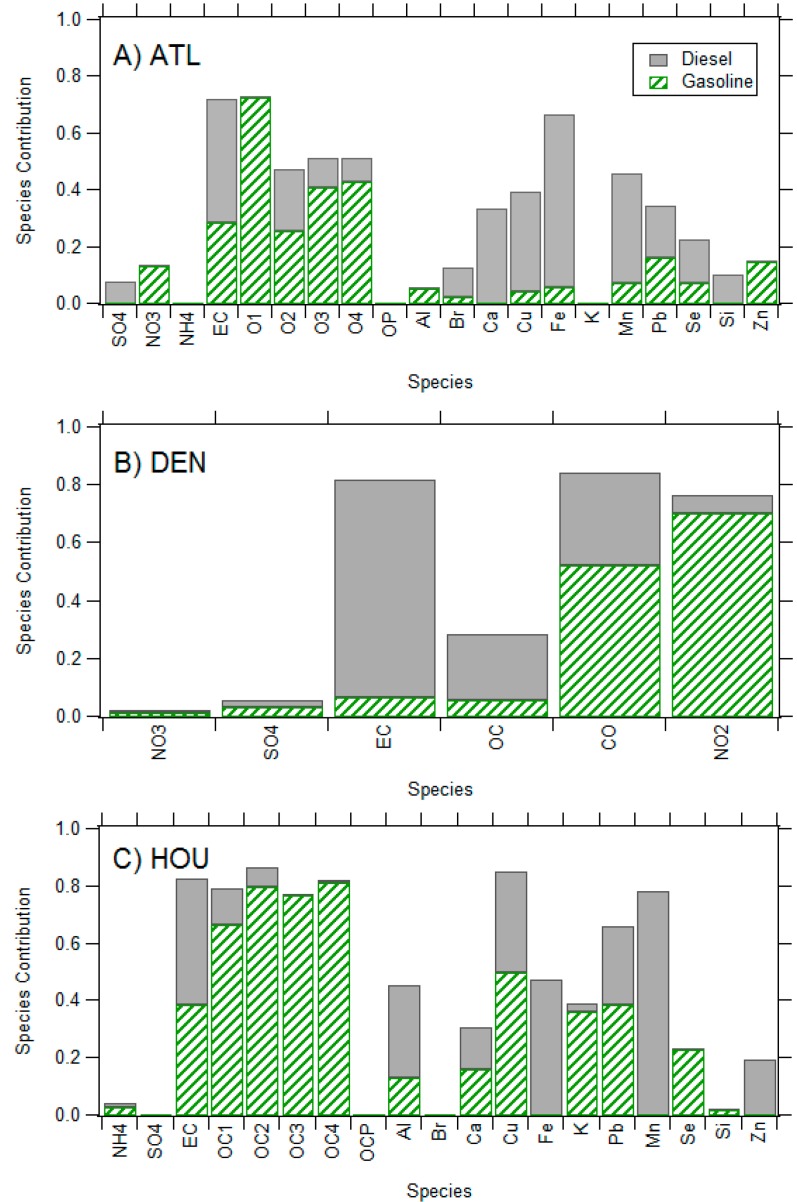
Chemical profiles of gasoline and diesel source apportionment factors in (**A**) Atlanta; (**B**) Denver; (**C**) Houston. Please view in color.

As seen in Figure 1, similar source profiles were observed for gasoline and diesel mobile source pollution across cities; however, some variation existed in contributions of PM and gaseous species present in each profile. EC was a major component of diesel profiles, whereas organic carbon was more prominent in gasoline profiles (with the exception of OC in Denver). Chemical signatures of road dust (Al, Ca), brake/tire wear (Fe, Cu), oil combustion (Zn) and ionic species (SO_4_^2^^−^, in Atlanta) were also apparent in both gasoline and diesel source profiles in Atlanta and Houston. Although similarities in gasoline and diesel profiles were evident across locations, the contribution of individual species often varied among different cities. For example, while potassium was a strong specie in the gasoline factors in Houston, it was not present in the gasoline factor in Atlanta.

#### 3.2.2. Emissions-Based Indicators

Annual mobile source emissions of CO, NO_x_, and EC (reported by the 2008 NEI [22]) showed substantial differences across Atlanta, Denver, and Houston (Figures S1–S3). In Atlanta, CO, NO_x_, and EC emissions were predominately from mobile sources, with mobile source emissions comprising more than 70% of total emissions of all three pollutants. Lower mobile source contributions (for all three pollutants) were observed in Denver and Houston. Annual CO and NO_x_ emissions from major air pollution source sectors (reported by the 2008 NEI [22]) demonstrate that non-mobile sources, such as EGUs, industrial combustion, and residential heating, likely contribute a significant portion of CO and NO_x_ emissions in Denver and Houston (Figures S2 and S3).

**Figure 2:**
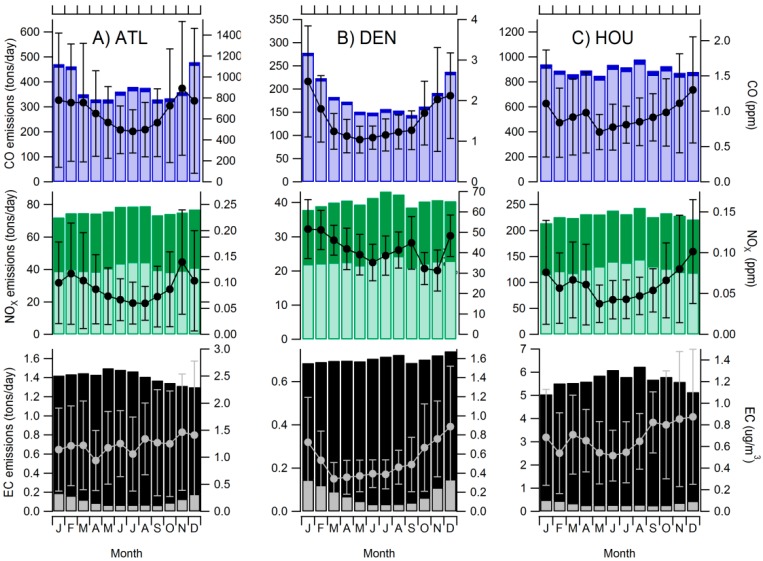
Monthly emissions for gasoline (lighter shade) and diesel (darker shade) mobile sources estimated by MOVES. Mean ambient concentrations observed at the central-site monitors are plotted (markers) with standard deviation in whiskers.

As shown in Figure 2, mobile source emissions of CO, NO_x_, and EC are associated with different portions of traffic pollution (diesel *vs.* gasoline). Across cities, NO_x_ was emitted in nearly equal proportions from both types of vehicles, while the majority (>80%) of CO and EC was emitted by gasoline and diesel vehicles, respectively. This suggests that NO_x_ likely provides an adequate representation of total mobile source pollution, while CO and EC tend to target gasoline and diesel emissions, respectively. Such results confirm the use of specific pollutant mixtures to describe gasoline, diesel and total fractions of mobile source pollution as defined in Equations (1)–(3). Additionally, no major month-to-month changes were observed in mobile source emissions and ambient pollutant concentrations, with the exception of CO and gasoline EC in Denver and Atlanta, which demonstrated increased values during the winter months, likely due to vehicular cold starts (Figure 2).

Table 4 presents mobile source fractions (α_X_) for CO, NO_x_, and EC used for IMSI quantification. Mobile source fractions for each pollutant (α_X_) were, for the most part, similar in Atlanta, Denver, and Houston; however, minor variations in these values reflect differences in mobile source emissions across locations. Based on our emissions analysis, mobile source fractions of CO, NO_x_ and EC were relatively the same across cities when estimating total mobile source emissions for IMSIEBs (approximately 33% for each pollutant), although mobile source fractions for CO and EC were slightly different in Houston. In contrast, CO (>62%) and EC (>55%) were the dominant pollutants used to estimate gasoline and diesel emissions in IMSIGVs and IMSIDVs, respectively. Notably, low NO_x_ mobile source contributions were observed across all indicators in Denver, compared to higher contributions in Atlanta and Houston. Such low NO_x_ mobile source fractions are largely attributed to significant non-traffic emissions from EGUs in Denver (Figures S1 and S3). For EC, lower mobile source fractions were observed in Houston for both IMSIDV and IMSIEB, consistent with a low mobile-to-total EC fraction demonstrated in Figure S1.

**Table 4:** Mobile source fractions for emissions-based indicators in three urban areas.

Urban Area	Type of Mobile Source Pollution	EC	NO_x_	CO
Atlanta, GA	Diesel	0.64	0.36	*
	Gasoline	*	0.38	0.62
	Combined	0.32	0.36	0.32
Denver, CO	Diesel	0.70	0.30	*
	Gasoline	*	0.32	0.68
	Combined	0.33	0.29	0.37
Houston, TX	Diesel	0.55	0.44	*
	Gasoline	*	0.35	0.65
	Combined	0.22	0.38	0.40

Note: * Not applicable.

### 3.3. Temporal Analysis of Single Pollutant and Multipollutant Metrics

**Figure 3:**
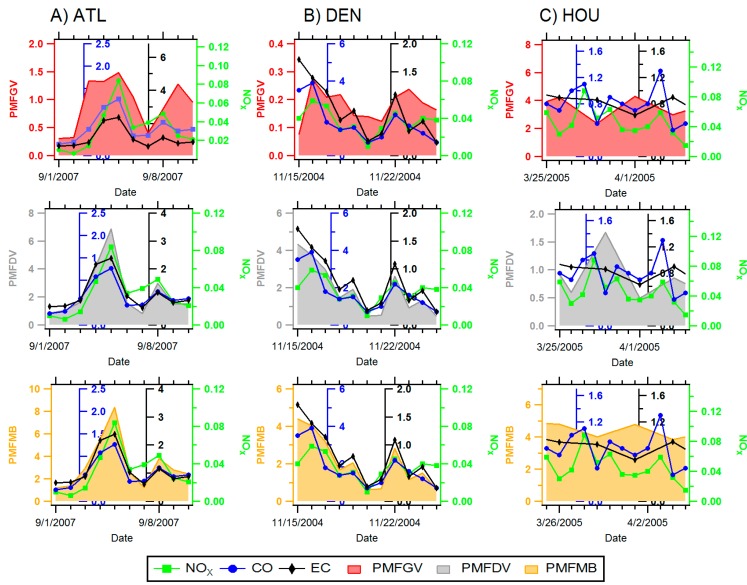
Time-series plot of single-pollutant (CO-blue, NO_x_-green, EC-black) and PMF mobile source factors (PMFGV (gas)-red shading, PMFDV (diesel)-gray shading, PMFMB-orange shading) in Atlanta, GA, Denver, CO and Houston, TX. Please view in color.

**Figure 4:**
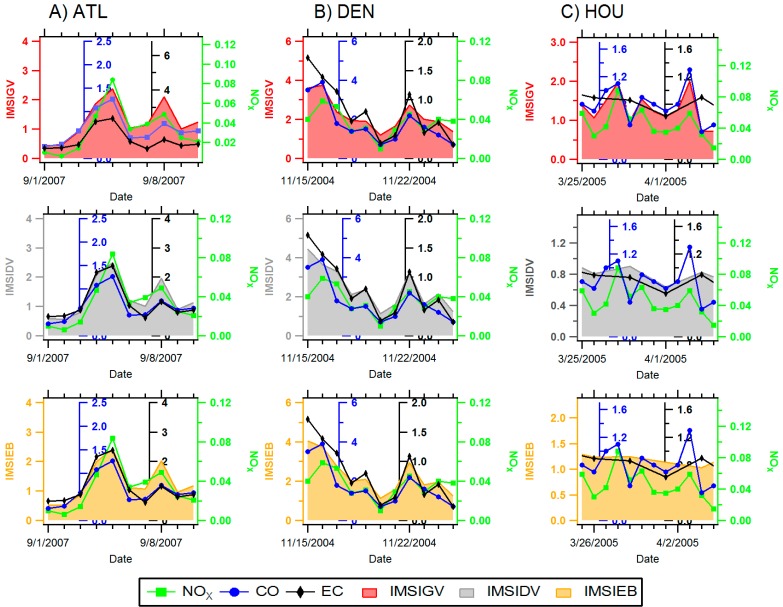
Time-series plot of single-pollutant (CO-blue, NO_x_-green, EC-black) and integrated mobile source indicators (IMSIGV (gas)-red shading, IMSIDV (diesel)-gray shading, IMSIEB-orange shading) in Atlanta, GA, Denver, CO and Houston, TX. Please view in color.

**Figure 5:**
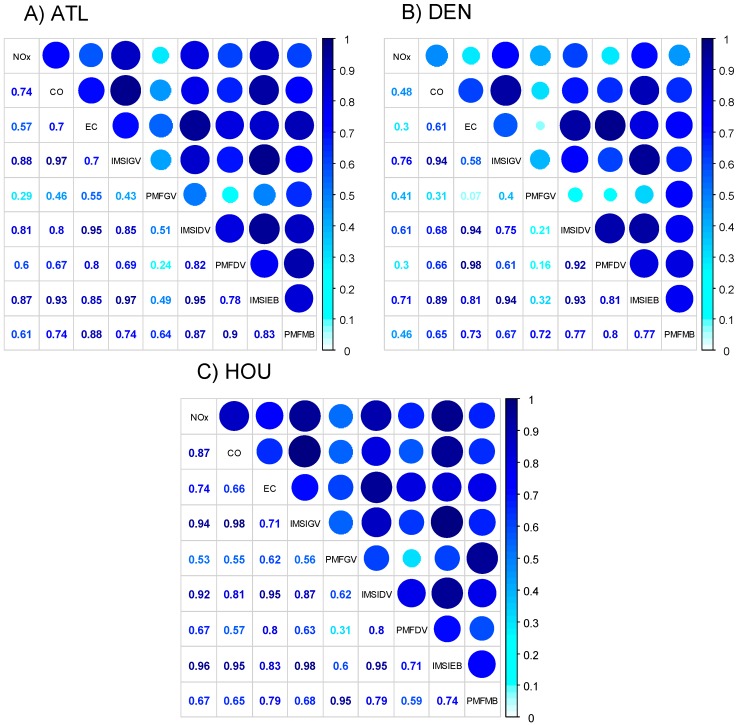
Pearson R for temporal correlations of central-site single pollutant and multipollutant indicators in (**A**) Atlanta, GA; (**B**) Denver, CO; (**C**) Houston, TX. The shading of the coefficient value and markers indicate the strength of Pearson correlation. Please view in color.

While substantial temporal variability was observed in central-site single pollutant metrics in each urban location, single pollutants tended to track one another on a day-to-day basis (Figure 3 and Figure 4). Pearson correlations presented in Figure 5 demonstrate that single pollutants (CO, NO_x_, and EC) were moderately correlated with one another (*r* generally > 0.6) in each city, reflecting a similar source among these pollutants, in this case, mobile sources. An exception to this trend was NO_x_ in Denver, which showed lower correlations with both CO (*r* = 0.48) and EC (*r* = 0.3). Relatively low correlations between NO_x_ and traffic co-pollutants likely point to the impact of non-traffic sources to ambient levels of NO_x_ in this location.

Correlations between central-site, single pollutant and multipollutant metrics were generally similar in different cities. In all cities, multipollutant gasoline metrics (IMSI-GV and PMF-GV) were more strongly correlated with CO (*r*: 0.31–0.93) compared to EC, while multipollutant diesel metrics IMSI-DV and PMF-DV) were better correlated with EC (*r*: 0.8–0.99) compared to CO (Figure 3, Figure 4 and Figure 5). Low correlations between CO and the PMF-GV in both Denver and Atlanta are notable. Alternatively, multipollutant indicators representing total mobile source pollution were, for the most part, strongly correlated with all three single pollutant traffic indicators (CO, NO_x_, and EC; *r* = 0.46–0.96). When comparing correlations between single pollutants and different types of multipollutant metrics, single pollutants typically showed higher correlations with IMSIs than PMF factors, with the exception of multipollutant diesel factors (IMSIDV and PMFDV), which showed similar Pearson correlations with EC.

In general, moderate to strong correlations were observed among multipollutant metrics in different locations (*r* = 0.61–0.98), aside from the source apportionment gasoline factor (PMFGV), which was generally poorly correlated with other multipollutant metrics (*r* = 0.16–0.95). In each location, individual IMSIs were well correlated with other IMSIs (*r* > 0.75), while moderately correlated with PMFMB and PMFDV factors (*r* > 0.61). Though PMFDV generally correlated with other PMF-derived factors, PMFGV generally did not correspond well to PMFDV metrics (*r* = 0.16–0.31).

### 3.4. Inter-Site Spatial Comparisons of Metrics in Different Locations

**Figure 6:**
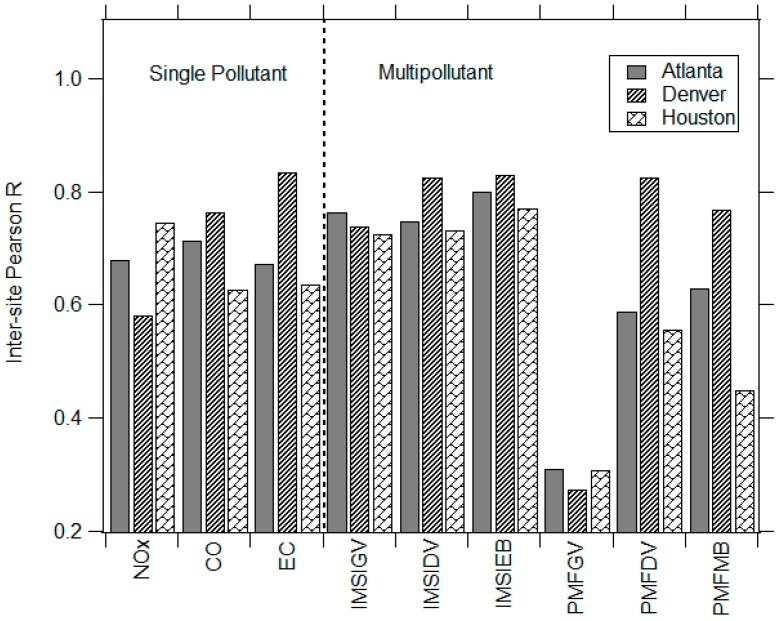
Pearson R values for inter-site spatial correlation of single pollutants, integrated mobile source indicators (IMSIs) and positive matrix factorization (PMF) factors in each urban area.

Inter-site spatial comparisons of single pollutant metrics (*i.e.*, comparison of individual daily metric values at a central-site *vs.* secondary site) in Figure 6 show that correlation coefficients for a given pollutant vary across cities, such that a high inter-site correlation in one city may not necessarily be observed in another city. In most urban areas, at least one single pollutant metric had relatively weak correlations between sites. In Denver, lower NO_x_ inter-site correlations were observed (*r* = 0.58) compared to CO and EC (*r* > 0.75). Spatial correlations in Houston were lower for CO (*r* = 0.63) and EC (*r* = 0.63), while NO_x_ showed higher correlations (*r* = 0.75). In Atlanta, spatial correlations were similar for all single pollutant metrics. Weak spatial correlations, such as those observed in Denver and Houston, may reflect the impact of local, non-traffic sources at different sites.

Across cities, moderate to high inter-site correlations were often observed in both IMSI and PMF factors; however, higher correlations were generally associated with IMSIs (*r* = 0.7–0.85) compared to PMF factors (*r* = 0.27–0.82) (Figure 6). The most notable contrast in inter-site comparisons of IMSIs and PMF factors was observed in gasoline-based metrics. In all three areas, poor spatial correlations were consistently observed in PMFGV (*r* < 0.3) compared to much higher correlations in IMSIGVs (*r* > 0.7). On the other hand, inter-site correlations associated with diesel and total mobile sources (IMSIDV, IMSIEB, PMFDV, PMFMB) exhibited only minor differences and were higher (*r* = 0.45–0.83).

Comparisons of spatial trends among single pollutant and multipollutant metrics demonstrated that IMSIs were more strongly correlated between two sites than single pollutants; while PMF inter-site correlations were neither consistently higher nor lower than single pollutant correlations. (Figure 6). For example, inter-site correlations among IMSIEB ranged from 0.77 to 0.83 in the cities, while NO_x_ exhibited lower correlation coefficients (*r* = 0.58–0.75). Similarly, inter-site comparisons among IMSIGV (*r* > 0.73) and IMSIDV (*r* > 0.73) were greater than CO (*r* > 0.44) and EC (*r* > 0.64), respectively. Less consistent trends were observed in inter-site comparisons among source apportionment factors and single pollutants. In all three cities, PMFGV was less spatially correlated than CO; while correlations for PMFDV and EC were similar.

## 4. Discussion

The goal of our study was to apply IMSIs to multiple locations and then compare them to widely used, traffic-related single pollutant (CO, NO_x_, EC) and multipollutant metrics (source apportionment factors) to evaluate the utility of IMSIs in future human health studies. Our work suggests IMSIs provide a robust, uniform method for estimating traffic pollution in different locations with varying emissions, topographical features, and meteorological behavior. Additionally, IMSIs appear to be comparable to other conventional single pollutant and multipollutant traffic metrics. Overall, our study supports initial findings by Pachon *et al.* [21] emphasizing the value of IMSIs for air quality and health studies.

Based on our spatial and temporal analysis, IMSIs tend to be more transferrable to multiple geographical locations and to multiple sites within an urban area than single pollutant indicators. While IMSIs appear to be good traffic surrogates regardless of location, no individual single pollutant indicator (CO, NO_x_, EC) is considered a consistent surrogate across multiple locations. In each urban area of this study, at least one of the single pollutant measures appears to inadequately represent traffic impacts. For example, while NO_x_ is found to be a reasonable surrogate in Atlanta and Houston, it does not appear to effectively represent traffic impacts in Denver due to the fact it has lower correlations with other mobile source indicators, a low inter-site correlation, and has a relatively low mobile source emissions contribution (Figure S1). These results suggest that ambient NO_x_ in Denver represents not only traffic pollution, but also represents variability in non-traffic sources, such as power plants. On the other hand, IMSIs were strongly correlated between multiple urban sites and tracked well with common traffic pollutants in every urban area of this study. Together, these results indicate that single pollutants can be influenced by local, non-traffic emissions, which in turn, affect their ability to serve as traffic surrogates in certain locations. Such local, non-traffic impacts on single pollutants may be enhanced if the sampling monitor is within close proximity to or directly downwind from major non-traffic sources. Local, non-traffic emissions may not have as large of an impact on comprehensive metrics, such as IMSIs, thus, making IMSIs more readily transferrable to different locations than single pollutants.

This transferability makes IMSIs appropriate for application in multicity air quality and epidemiologic studies that compare source impacts across multiple cities using a uniform approach. As demonstrated in our study, IMSIs provide a standard approach for estimating traffic pollution (as defined by Equations (1)–(3)) that relies on local emissions information and routinely measured pollutant data. Alternatively, source apportionment of mobile sources tends to vary by location, often yielding results that are difficult to compare across cities. In this study, different pollutants were used for source apportionment analyses to adequately resolve mobile source pollution in Atlanta, Denver, and Houston, resulting in city-to-city variations in mobile source profiles. Comparing mobile sources with different chemical profiles between cities is not always straightforward. For instance, in health studies, it is challenging to directly link a particular health effect to a source in one city if the characteristics of a similar source vary in another city [33]. Overall, these results demonstrate the challenges in applying source apportionment to multi-city studies.

Another feature of IMSIs is their ability to provide a straightforward approach for separating gasoline and diesel mobile source contributions, which is beneficial in applications intending to identify specific pollutant mixtures responsible for health effects. Our results showed that in each city both gasoline and diesel IMSIs exhibited strong correlations between two urban sites and with traffic co-pollutants, indicating that IMSIs can resolve different portions of traffic pollution over large geographical scales with low spatial error. Single pollutant and source apportionment results were less consistent. For example, in Houston, lower inter-site correlations observed in both EC (diesel indicator) and CO (gasoline indicator) coupled with emissions information indicating moderate to large non-traffic contributions, suggest these pollutants may not fully capture diesel or gasoline mobile source pollution by themselves. Additionally, while PMFDV factors appeared to capture urban-scale diesel trends in every city, PMFGV factors were less correlated over similar spatial scales, thus, are likely associated with relatively large amounts of spatial error.

The ability of source apportionment (PMF) to effectively resolve pollution from diesel vehicles, but not necessarily gasoline vehicles, may be in part due to the fact that PMF relies on multiple species, with varying degrees of measurement uncertainty and ambient concentration levels, to separate different mixtures. Sources linked to species measured near instrument detection limits or measured with large uncertainties can be difficult to identify using source apportionment. In this study, PMF relied heavily on EC, a well measured pollutant present at detectable levels, to identify diesel factors. On the other hand, PM species such as organic carbon (OC) and trace metals, that are measured using varying techniques, or are present at trace levels, are primarily used to resolve gasoline factors. The results of this study, like other studies, highlight the challenges in separating gasoline and diesel fractions of mobile source pollution using standard source apportionment methods [17,19,20,34].

Although our study sheds light on spatial and temporal characteristics of various mobile source indicators, several limitations are inherent with this study that should be considered when interpreting the results. First, our spatial analysis only includes two sampling sites in each city, a central site and a secondary site. While a comprehensive analysis using multiple sites could provide more information on urban-scale spatial variability and exposure error of different metrics, the routine monitoring network often does not have multiple sites with necessary monitoring equipment, and hence data are not available in most cities. Second, the sampling frequency varied across pollutants and urban areas, which could have biased our spatial and temporal comparison among metrics. In some cities (Houston and Atlanta), EC was measured every 3rd or 6th day compared to gaseous species (CO, NO_x_) reported on a daily basis. Last, there is currently no approach to definitively measure source impacts to use as a reference point for estimating the traffic pollution impacts on exposure, which presents challenges in how results can be interpreted and methods can be compared. Here, we compared spatiotemporal characteristics of different metrics with one another to assess their similarities and differences.

Among the multipollutant metrics evaluated in this study, our results clearly demonstrate that both IMSIs and PMF factors can be reliable traffic surrogates in a variety of locations; however, the process of constructing each metric has some uncertainty that may hamper its utility in certain applications. For example, IMSIs rely on mobile source emissions estimates that are based on vehicle test data that may differ from actual emissions from in-use vehicles, particularly under different meteorological conditions. The estimates also rely on assumptions regarding the fleet mixture and driving conditions, which account for variability and uncertainty in the resulting emissions data. Furthermore, most publicly-accessible emissions data are only available on relatively coarse scales, both temporal (weeks to years) and spatial (county-level). Applying coarsely-resolved emissions to IMSIs assumes that mobile source contributions (*i.e.*, pollutant specific mobile source fractions (α_X_)) do not vary substantially over space or on a day-to-day basis, which presents some limitations. It is possible, however, to apply time-specific emissions to IMSIs resulting in different mobile source fractions for different periods of the study. This application is particularly ideal for longer-term studies that overlap with time periods of decreasing emissions due to fuel and vehicle controls.

Source apportionment, on the other hand, relies on pollutant measurements, some of which are poorly measured, highly uncertain or near the method detection limit, which often results in unrealistic source data that are difficult to interpret. As previously discussed, this uncertainty may have contributed to the relatively poor performance of the gasoline factors in our study. Source impacts (resolved by PMF) can also exhibit aberrant temporal variability lacking correspondence to local emissions and measurement data [31,35,36]. Moreover, in longer term studies, the use of PMF can also be complicated due to changes in source profiles over time that make it difficult to resolve specific gasoline and diesel profiles unless the data are broken down into separate time periods [37]. Last, due to similar temporal patterns among gasoline and diesel mobile source emissions, moderate correlations tend to exist between the two types of mobile source metrics, whether considering IMSI or PMF metrics. Such correlations do not necessarily reflect the inability of IMSIs or PMF to resolve different types of mobile source pollution, but may present challenges in attributing distinct health impacts to gasoline and diesel mobile sources. Overall, these combined uncertainties can have a profound effect on the quality of results in an air pollution or health study and should be considered by an investigator when designing a study.

Investigators should also consider which portion of the traffic mixture should be represented prior to selecting a metric, recognizing that traffic pollution is a complex mixture (of tailpipe and non-tailpipe emissions) and individual metrics likely represent this mixture differently. IMSIs, for example, primarily include information from pollutants in tailpipe exhaust and likely adequately capture this portion of the mixture. Alternatively, source apportionment can incorporate combustion pollutants as well as pollutants from brake/tire wear (e.g., Ba, Cu), secondary formation processes, and road dust (e.g., Fe, Al), which may allow for better resolution of non-exhaust traffic pollution than provided by IMSIs or single pollutant metrics. However, combining tailpipe and non-tailpipe pollutants in source apportionment may also result in factors that are difficult to directly link to traffic pollution. Using pollutants with unstable chemical properties (e.g., semivolatile pollutants: organic carbon) for source apportionment can bias results [38,39], especially across cities. For example, OC may be more volatile in Houston than in Denver due to meteorological factors, resulting in large differences among mobile source impacts and profiles in different locations.

We conclude by addressing the following question: “What can be gained by using multipollutant metrics instead of single pollutant metrics for mobile sources?” Previous scientific reviews have emphasized the importance of employing a multipollutant paradigm when evaluating air pollution health effects, but have also recognized the dearth of research on the application of multipollutant metrics in such studies [7,8,9,10,11]. To address this research gap, we compared a variety of single pollutant and multipollutant metrics to gain insight to their utility in future air pollution health effects research. Our study shows several advantages of using multipollutant metrics for mobile source pollution, such as improved transferability within and across cities, lower CVs, and generally greater inter-site correspondence. Additionally, prior studies using similar metrics in health studies have found associations with somewhat smaller uncertainties though virtually the same exposure response functions [21]. Although, in many cases, the difference between multipollutant and single pollutant metrics is small, suggesting that using advanced metrics may not necessarily translate to a better understanding of sources and/or health effects.

With respect to characterizing temporal variability, multipollutant metrics tend to decrease day-to-day fluctuations in mobile source impacts more than most single pollutant metrics, especially daily 1-h max metrics, as evidenced by lower CVs in multipollutant measures (Table 1). This dampening effect can have either a positive or negative impact on exposure and/or human health studies. For example, using multipollutant metrics may reduce the amount of variability due to sampling error, changes in seasonal concentrations, or non-traffic emissions, which likely impact single pollutants more than multipollutant metrics. This additional variability may not directly reflect mobile source pollution, thus, resulting in an added layer of exposure error in a health study. On the other hand, multipollutant metrics can disproportionately dampen or artificially smooth temporal variability to the point where such metrics underestimate actual variability in traffic pollution, which may partially reduce statistical power in detecting an exposure-health effect relationship. Such dampening effects on temporal characteristics appear to be more prominent in IMSIs, which utilize coarsely-resolved traffic emissions and normalized ambient concentration values, and could have positively biased inter-site correlations among IMSIs. Applying more fine-scale spatial emissions data may result in lower inter-site correlations. Additionally, some single pollutant metrics, such as 24-h average single pollutant measures, have similar CVs as IMSIs, making it difficult to evaluate which metric is more representative of traffic pollution. Clearly, there are specific drawbacks associated with the degree to which either single pollutant or multipollutant metrics characterize temporal variations in traffic; however, an effective indicator is one that incorporates an adequate amount of temporal variability while minimizing exposure error, which may not necessarily be multipollutant in nature.

Last, multipollutant metrics likely have an added degree of uncertainty associated with the process of combining several pollutant concentrations and other data into a metric [11], and failing to account for such uncertainty can potentially skew the exposure-health effect relationship (*i.e.*, by artificially narrowing confidence intervals around effect estimates) [40]. Kioumourtzoglou *et al.* [40] showed that uncertainty associated with source apportionment methodology can inflate standard error in mobile source effect estimates. An earlier study [21] showed similar relative uncertainties for IMSIs (0.51–0.68) and PMF-derived (0.26–0.67) mobile source factors and concluded that error associated with these methods largely stems from pollutant measurements, traffic emission estimates, copollutant covariance structures, and the ability of an individual multipollutant technique to identify mobile sources consistently. Based on these results, it is reasonable to speculate that IMSIs and PMF factors have similar impacts on health effect estimate uncertainty. On the other hand, the results of this study, as well as Pachon *et al.* [21], suggest that IMSIs have lower spatial error over large geographical scales compared to single pollutant or PMF-derived indicators. Low spatial error (associated with IMSIs) may counterbalance additional exposure error from constructing the IMSI, thus, indicating a potential advantage of using IMSIs over PMF. However, all metrics included in this study rely on central-site monitoring data, meaning that they may not adequately capture spatial variability and do not account for activity patterns, potentially leading to attenuated effect estimates [41]. Thus, it remains largely unclear how uncertainty associated with IMSIs or PMF will impact health estimates. Moving forward, it is important for researchers to constrain the effect of multipollutant metrics exposure on effect estimate uncertainty, in particular, how such uncertainty impacts the direction and magnitude of risk estimates.

## 5. Conclusions and Future Work

Overall, we successfully applied emission-based multipollutant indicators to urban areas with varying traffic emissions. These indicators appeared to be more transferrable to multiple geographic areas than traditional single pollutant and multipollutant metrics, with more consistent correlations among metrics and between sites. Therefore, IMSIs are well-suited for multi-city study designs interested in comparing source impacts and subsequent health effects across cities. However, in many cases, there were no major differences in the single pollutant and multipollutant metrics representing urban-scale traffic pollution, which suggests that some uncertainty exists regarding the benefits provided by multipollutant metrics (IMSIs and source apportionment factors) in air quality and human health studies.

Future work evaluating different types of single pollutant and multipollutant metrics in epidemiologic analyses will address remaining questions on the value of different metrics. In particular, studies comparing the effect of different types of metrics on exposure and health effect uncertainty will be most informative [42]. Additionally, extending these indicators to other sources beyond traffic is beneficial, especially to sources that are difficult to capture using standard single pollutant or multipollutant techniques and that have significant health implications (e.g., biomass burning). The use of concentration data from chemical transport models, such as the Community Multiscale Air Quality Model (CMAQ), can provide information on species that are not routinely measured, which in turn, will help to facilitate extending these indicators to new sources.

## Supplementary Files

Supplementary File 1
