# Supplementary material for: Comparing Multipollutant Emissions-Based Mobile Source Indicators to Other Single Pollutant and Multipollutant Indicators in Different Urban Areas
Source: Int J Environ Res Public Health. 2014 Nov 14;11(11):11727–52. doi: 10.3390/ijerph111111727 (PMC4245641; doi:10.3390/ijerph111111727)
Supplement: Supplementary File 1 [file ijerph-11-11727-s001.pdf]

Supplemental Information

Comparing Multipollutant Emissions-Based Mobile Source Indicators to Other Single Pollutant and Multipollutant Indicators in Different Urban Areas

---

Supplemental Text

1. PMF methods
2. PMF results
3. References

Tables

1. Species and settings used in PMF analysis

Figures

1. NEI 08 annual on-road mobile source emissions in Atlanta, Denver, and Houston
2. NEI 08 CO Emission Estimates for Tier 1 Source Sectors in Atlanta, Denver, and Houston
3. NEI 08 NO_x Emission Estimates for Tier 1 Source Sectors in Atlanta, Denver, and Houston

Supplemental Text

PMF Methods

Positive Matrix Factorization (PMF) is a multivariate factor analysis tool that decomposes a matrix of speciated sample data into a factor contribution matrices and a factor profile matrices. The goal of PMF is to identify the number of factors *p*, the species profile *f* of each source, and the source contribution *g* to each sample that solves the chemical mass balance between measured species concentrations (x_{ij}) and source profiles (Equation (S1) below):

$$x_{ij} = \sum_{k=1}^{p} g_{ik} f_{kj} + e_{ij} \quad \text{(S1)}$$

where e_{ij} is the residual for each sample/species.

The method is summarized briefly here and described in more detail elsewhere-[1,2]. PMF uses a constraint that no sample can have a significantly negative source contributions. Each sample data point is weighted with a user-provided uncertainty associated with the sample concentration. PMF then attempts to minimize the objective function Q (Equation (S2) below) based upon the estimated or adjusted sample uncertainties u_{ij}.

$$Q = \sum_{i=1}^{n} \sum_{j=1}^{m} \left[\frac{x_{ij} - \sum_{k=1}^{p} g_{ik} f_{kj}}{\mu_{ij}} \right]^2 \quad \text{(S2)}$$

In this analysis, source apportionment was conducted using EPA PMF 5.0 [3] which utilizes the multilinear engine (ME-2) to solve Equation (S2). New features of EPA PMF 5.0 include two additional error estimation methods for factor solutions and the ability to use constraints for source contributions and profiles [3,4]. Twenty-four hour, speciated $PM_{2.5}$ data as part of the CSN/STN for Deer Park, TX and Aldine, TX were retrieved from the EPA's Air Quality System (AQS) Database for the years 2003–2006. EPA PMF requires both a concentration and uncertainty data files as input. A total of 20 species were included for the Deer Park site and 18 species for the Aldine site. It should be noted that for the Aldine site the nitrate data was not available and pyrolytic carbon (OP) was not used as almost 50% of the data were either missing or below detection. The uncertainty values were calculated using Equation (S3) as described in [5].

$$UNC_{ij} = \frac{MDL_{ij}}{3} + k_j * x_{ij} \quad \text{(S3)}$$

where MDL is the method detection limit, x_{ij} is the concentration of species j measured for sample i, and k_j is fraction estimated for each species j using uncertainty versus concentration plots. Missing data was replaced using the median value as the concentration and 4 times the median as the uncertainty [6]. A complete summary of species and settings used in PMF for each site is summarized in Table 1 as recommended by [4]. Factor solutions were evaluated using the bootstrap (BS) and displacement (DISP) error estimation tools in EPA PMF. For each site, 100 bootstraps were run using a random seed and minimum correlation value of 0.6 for all species at both sites. All strong species were included in the DISP run for each site. No constraints were applied to the data sets.

PMF Results

A six factor solution was determined for the Deer Park CSN site: diesel (5.4% by mass), secondary nitrate (11.4%), secondary sulfate (42.4%), gasoline (8.2%), biomass burning (19.5%), and soil (13.1%). The gasoline profile contained elevated amounts of OC (OC1 through OC4) and EC as well as components associated with tire wear, brake wear, and road dust (e.g., Zn, Pb, and Cu). The diesel profile contained high levels of EC and OP in addition to road dust and brake wear signatures (e.g., Al, Cu, Fe, and Mn). A six factor solution was determined for the Aldine STN site: gasoline (25% by mass), Zn-rich (0.6%), soil (12.2%), secondary sulfate (38.6%), diesel (7.7%), and biomass burning (16.0%). This gasoline profile contained high levels of OC (OC1 through OC4) and EC as well as components associated with road dust and brake wear (e.g., Ca, Cu, and Pb). The diesel profile contained high levels of EC along with components associated with brake wear and road dust (e.g., Al, Cu, Fe and Mn). The bootstrap and displacement error estimation results suggested that the factor solutions are feasible for both sites. PMF run diagnostics for Deer Park and Aldine are reported in the supplement (Table S1) as recommended by [4].

Table S1. Species and settings used in PMF analysis.

Site	Deer Park, TX (2003–2006)	Aldine, TX (2003–2006)
N Samples	318	358
Strong Species	Br, Ca, Fe, Mn, Si, Zn, K, NH4, SO4, EC	Br, Ca, Fe, Mn, Si, Zn, NH4, SO4, EC, OC1, OC2, OC3, OC4
Weak Species	Mass (total variable), Al, Cu, Pb, Se, OC1, OC2, OC3, OC4, OP	Mass (total variable), Al, Cu, Pb, Se, K
Sample Dates Removed	16 January 2005(Measured mass >25% below reconstructed mass *)<br>13 October 2005 (High Zn)<br>4 July 2006 (High K due to fireworks)	1 January 2004, 5 July 2004, 26 December 2004, 1 January 2005, and 4 July 2006 (High K due to fireworks)<br>24 October 2006 (High Br)<br>28 August 2003, 9 May 2004, 14 December 2004, 3 February 2005 (Measured mass >25% below reconstructed mass *)
Number of Factors	6	6
Robust Mode	Yes	Yes
Number of BS	100	100
Minimum correlation value for BS	0.6	0.6
Species included in DISP	All strong species	All strong species

* Reconstructed Mass = (2.14 × Si) + (1.4 × Ca) + (1.43 × Fe) + (1.2 × K) + (1.24 × Zn) + (1.8 × Cl) + (1.38 × SO_4) + (1.29 × NO_3) + (1.8 × OC) + EC.

Figure S1. Annual on-road mobile source emissions in Atlanta, Denver, and Houston.

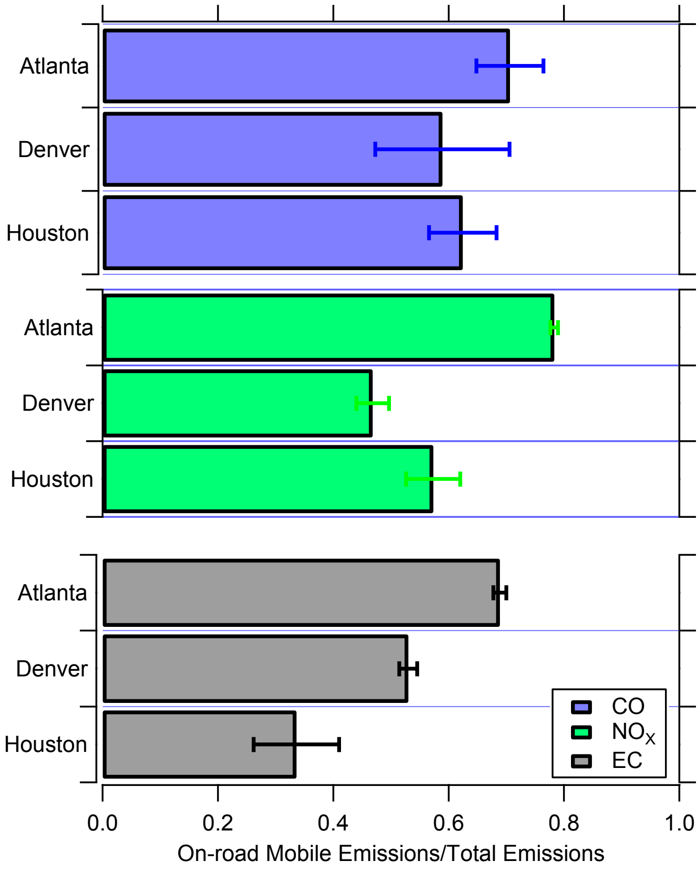

Figure S2. NEI 08 CO Emission Estimates for Tier 1 Source Sectors in (**A**) Atlanta; (**B**) Denver; and (**C**) Houston.

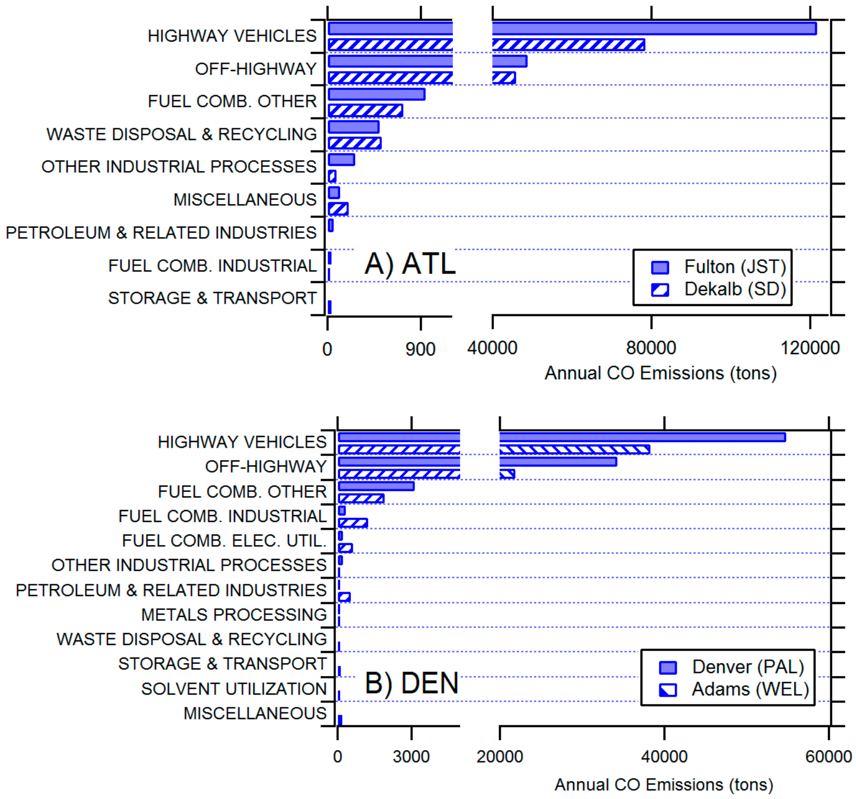

Figure S2. *Cont.*

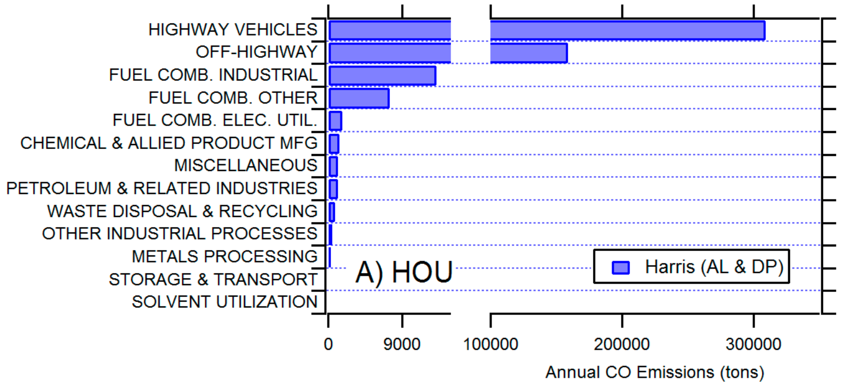

Figure S3. NEI 08 NO_x Emission Estimates for Tier 1 Source Sectors in Atlanta, Denver, and Houston.

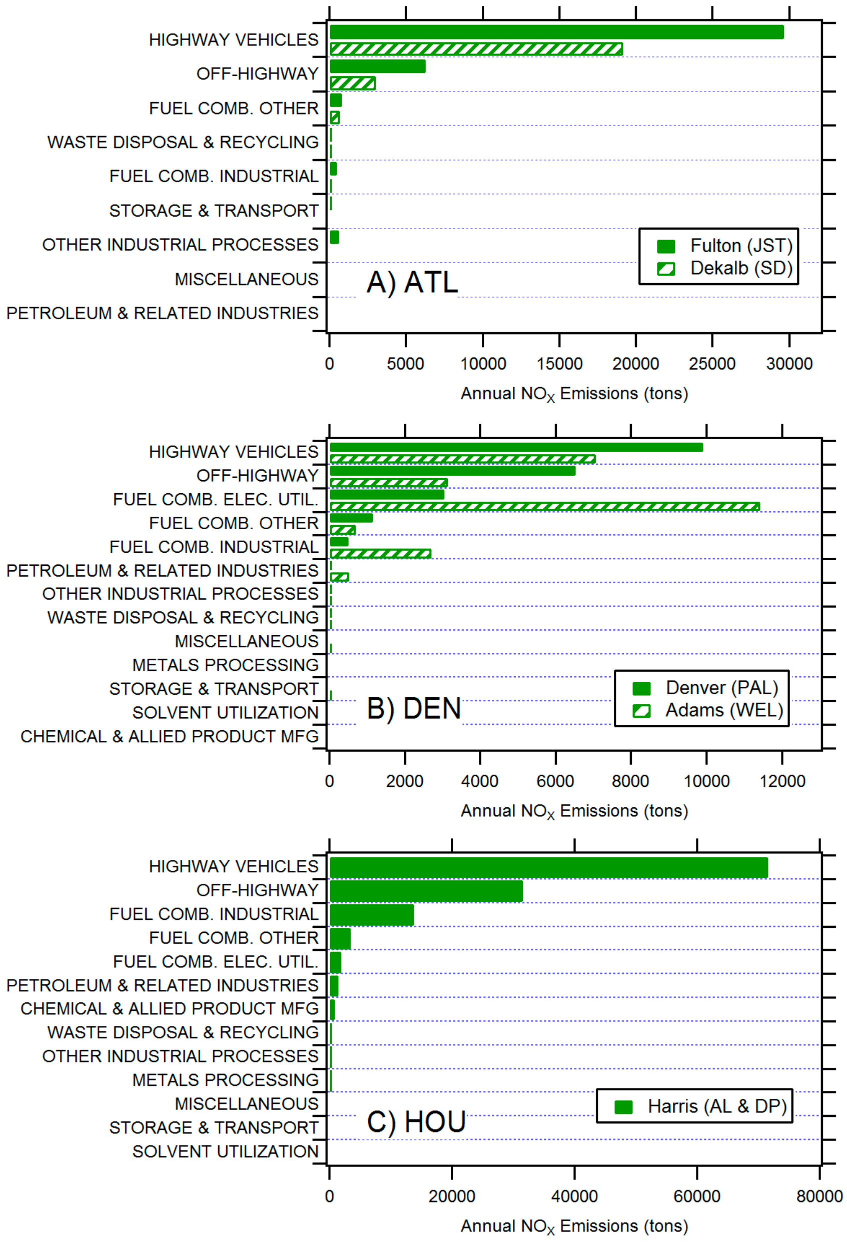

References

1. Paatero, P.; Tapper, U. Positive matrix factorization: A non-negative factor model with optimal utilization of error estimates of data values. *Envirometrics* **1994**, *5*, 111–126.
2. Paatero, P. Least squares formulation of robust non-negative factor analysis. *Chemom. Intell. Lab. Syst.* **1997**, *37*, 23–35.
3. Norris, G.; Duvall, R.; Brown, S.; Bai, S. *EPA Positive Matrix Factorization (PMF) 5.0 Fundamentals and User Guide*; Office of Research and Development, U.S. Environmental Protection Agency: Washington, DC, USA, 2014.
4. Brown, S.G.; Eberly, S.; Paatero, P.; Norris, G.A. Methods for estimating uncertainty in PMF solutions: Examples with ambient air and water quality data. *Atmos. Meas. Tech.* **2014**, *7*, 781–797.
5. Kim, E.; Hopke, P.K.; Qin, Y. Estimation of organic carbon blank values and error structures of the speciation trends network data for source apportionment. *J. Air Waste Manage. Assoc.* **2005**, *55*, 1190–1199.
6. Polissar, A.V.; Hopke, P.K.; Paatero, P.; Malm, W.C.; Sisler, J.F. Atmospheric aerosol over Alaska: 2. Elemental composition and sources. *J. Geophys. Res.* **1998**, *103*, 19045–19057.

© 2014 by the authors; licensee MDPI, Basel, Switzerland. This article is an open access article distributed under the terms and conditions of the Creative Commons Attribution license (http://creativecommons.org/licenses/by/4.0/).
